# Two new species of the genus *Andixius* Emeljanov & Hayashi from China (Hemiptera, Fulgoromorpha, Cixiidae)

**DOI:** 10.3897/zookeys.739.13043

**Published:** 2018-02-22

**Authors:** Yan Zhi, Lin Yang, Pei Zhang, Xiang-Sheng Chen

**Affiliations:** 1 Institute of Entomology, Guizhou University, Guiyang, Guizhou, 550025, P.R. China; 2 The Provincial Special Key Laboratory for Development and Utilization of Insect Resources of Guizhou, Guizhou University, Guiyang, Guizhou, 550025, P.R. China; 3 Laboratory Animal Center, Guizhou Medical University, Guiyang, Guizhou, 550025, P.R. China; 4 Xingyi Normal University for Nationalities, Xingyi, Guizhou, 562400, P.R. China

**Keywords:** Fulgoroidea, morphology, planthopper, taxonomy

## Abstract

Two new cixiid planthopper species: *Andixius longispinus* Zhi & Chen, **sp. n.** and *A. trifurcus* Zhi & Chen, **sp. n.** are described and illustrated from China (Yunnan Province) to provide the genus with four species in total. The generic characteristics are redefined. A key based on male genitalia to the four known species of this genus and a map of their geographic distributions are provided.

## Introduction

Emeljanov and Hayashi (2007) established the cixiid planthopper genus *Andixius* with two species: *Andixius nupta* Emeljanov & Hayashi, 2007 (as its type species) from Japan and *Andixius venustus* (Tsaur & Hsu, 1991) (previously placed in the genus *Brixia* Stål, 1856) from China. This genus belongs to the tribe Andini of subfamily Cixiinae (Hemiptera: Cixiidae). Characteristics of the tribe Andini are the downward bend of the apical part of segment XI which is usually longer than the anal style, and also anastomoses in one point (as if a crossing) of veins MP and CuA in the hindwing. So far, this genus includes only two species (Tsaur and Hsu 1991; Emeljanov and Hayashi 2007).

Herein, two new species: *Andixius longispinus* Zhi & Chen, sp. n. and *A. trifurcus* Zhi & Chen, sp. n. are described and illustrated from Yunnan province, China. The genus *Andixius* now contains four species, including three from China. A key based on male genitalia to all known species is provided as well as a map of their geographic distributions.

## Materials and methods

The morphological terminology and measurements follow Tsaur et al. (1988) and Löcker et al. (2006). Body length was measured from apex of vertex to tip of forewing; vertex length was measured the median length of vertex (from apical transverse carina to tip of basal emargination). External morphology and drawings were done with the aid of a Leica MZ 12.5 stereomicroscope. Photographs of the types were taken with KEYENCE VHX-1000 system. Illustrations were scanned with CanoScan LiDE 200 and imported into Adobe Photoshop CS7 for labelling and plate composition. The dissected male genitalia are preserved in glycerine in small plastic tubes pinned together with the specimens.

The type specimens examined are deposited in the Institute of Entomology, Guizhou University, Guiyang, Guizhou Province, China (**IEGU**).

## Taxonomy

### Andixius

Taxon classificationAnimaliaHemipteraCixiidae

Emeljanov & Hayashi, 2007

Andixius Emeljanov & Hayashi, 2007: 127.

#### Type species.

*Andixius nupta* Emeljanov & Hayashi, 2007, original designation.

#### Diagnosis.

The distinctive characters proposed by Emeljanov and Hayashi (2007) are modified as follows: head including eyes distinctly narrower than pronotum. Apical transverse carina of vertex weak and low, meeting the main part of frons at slightly obtuse angle or arc. Subapical carina of vertex as strong as conjunct lateral carinae. A small almost quadrangular fossette between vertex and frons. Vertex narrowest at apex, widened to base, disc arcuately and deeply excavated, lateral carinae strongly elevated. Lateral carinae of frons and postclypeus foliate, directed forward; carinae of posclypeus slightly lower than those of frons. Lower part of frons with convex disc separated from lateral carinae; its upper part deeply trough-like. Frontoclypeal suture slightly arched dorsally at middle. Middle ocellus apart from postclypeus. Middle carina of frons only in lower part or absent. Clypeus with distinct median carina. Frontoclypeus compressed, without lateral carinae. Rostrum long, extended considerably beyond hind coxae. Antennae medium-sized; pedicellum rounded conical and isodiametric. Pronotum short, with anterior margin straight and posterior margin deeply emarginated in an angle; intermediate carinae of pronotum encircling eyes from behind and below. Lateral carinae of pronotum between eye and tegula, separating paranotal lobes of pronotum from upper part behind postocular carinae. Tegmina long, tectiform, gradually but distinctly expanded towards end, rounded at apex, ScR (ScRA and RP) forming a short common stalk. Legs simple, fore coxae without angular apical lobe, hind tibia with six apical spines.

#### Remarks.

This genus can be easily distinguished from other genera of Andini by the following characters: forewings without trifid branching of ScRA, RP and M near basal cell, ScR (ScRA and RP) forming a short common stalk. Legs simple, fore coxae without angular apical lobe.

#### Distributions.

China, Japan.

**Checklist and distributions of species of *Andixius* Emeljanov & Hayashi**

*A. longispinus* Zhi & Chen, sp. n., China (Yunnan).

*A. nupta* Emeljanov & Hayashi, 2007, Japan (Ryukyu).

*A. trifurcus* Zhi & Chen, sp. n., China (Yunnan).

*A. venustus* (Tsaur & Hsu, 1991), China (Taiwan).

#### Key to species of *Andixius* (males)

**Table:** 

1	Periandrium with an expanded semi-enclosed structure around the left side and ventral margin of periandrium. Ventral margin of the expanded structure trifurcated into three long processes (Figs 25–28)	***A. trifurcus* sp. n.**
–	Periandrium without expanded semi-enclosed structure	**2**
2	Left side of periandrium with a bifurcate process (Emeljanov and Hayashi 2007: 130, figs 11–13)	***A. nupta***
–	Left side of periandrium without process or the process on left side of periandrium not bifurcated	**3**
3	Ventral margin of periandrium without process, right side of flagellum with a large bifurcate process (Hsu and Stalle 1991: 66, fig. 33 (D–F))	***A. venustus***
–	Ventral margin of periandrium with a projection, of which basal 1/3 longitudinally and distal 2/3 horizontally extended, flagellum with two “simple” processes, not bifurcate (Figs 13–16)	***A. longispinus* sp. n.**

### Andixius longispinus

Taxon classificationAnimaliaHemipteraCixiidae

Zhi & Chen sp. n.

http://zoobank.org/E3AD0FA5-2544-47DD-8ADF-0609D2D9167D

Figs 1–2 , 5–16

#### Type material.

Holotype: ♂, **China**: Yunnan, Lushui County, Pianma Town (26°N, 98°36'E), 16 August 2000, Xiang-Sheng Chen; paratypes: 1♂1♀, same data as holotype.

#### Description.

Body length: male 6.2–6.5mm (*N* = 2), female 7.2 mm (*N* = 1); forewing length: male 5.4–5.5 mm (*N* = 2), female 6.3 mm (*N* = 1).

**Figures 1–4.**
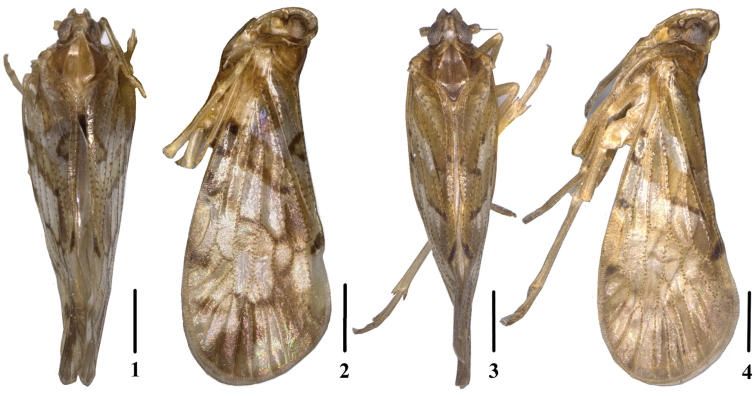
**1–2.** *Andixius longispinus* sp. n., male. **1** Dorsal view **2** Lateral view **3–4.** *Andixius trifurcus* sp. n., male **3** Dorsal view **4** Lateral view. Scale bars: 1.0 mm.

*Coloration.* General color yellowish brown (Figs 1–2). Eyes brown, ocelli faint yellow, semi-translucent. Antenna, vertex, face and rostrum generally yellowish brown. Pronotum with discal area yellowish brown and lateral areas dark yellowish brown. Mesonotum with areas between lateral carinae yellowish brown, lateral areas brown. Forewing semi-translucent, costal vein with 3 small spaced dark brown spots; slightly behind stigma, near claval fork and behind clavus with an irregular tan spot respectively, basal and middle part of forewings with two inner oblique brown stripes; apical half of wing with brown patches. Hind tibiae and ventral abdomen yellowish brown.

**Figures 5–16.**
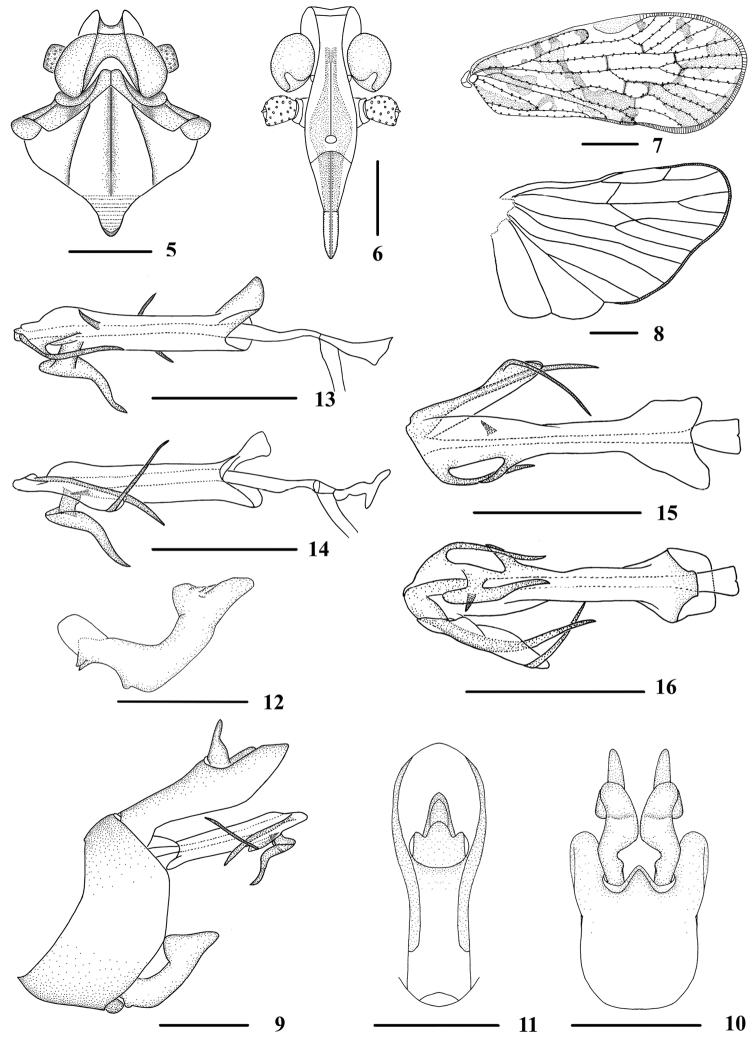
*Andixius longispinus* sp. n., male **5** Head and thorax, dorsal view **6** Face, ventral view **7** Forewing **8** Hindwing **9** Genitalia, lateral view **10** Pygofer and genital styles, ventral view **11** Anal segment, dorsal view **12** Genital styles, lateral view **13** Aedeagus, right side **14** Aedeagus, left side **15** Aedeagus, dorsal view **16** Aedeagus, ventral view. Scale bars: 0.5 mm (**5–6, 9–16**); 1.0 mm (**7–8**).

*Head and thorax*. Vertex (Figs 1, 5) 0.8 times longer than wide; anterior margin nearly straightly, posterior margin V-shaped recessed, median carina absent. Frons (Fig. 6) 2.6 times as long as wide. Pronotum (Figs 1, 5) 1.8 times longer than vertex; posterior margin obtuse-angled. Mesonotum 1.4 times longer than pronotum and vertex combined. Forewing (Figs 2, 7) 2.2 times longer than wide, with twelve apical cells and six subapical cells. Hind tibia with six lateral spines, usually small; chaetotaxy of hind tarsi: 7/6–7, 2^nd^ hind tarsus with 0–3 platellae.

*Male genitalia*. Pygofer (Figs 9–10) symmetrical, dorsal margin shallowly concave and U-shaped ventrally, widened towards apex, slightly concaved medially; in lateral view, lateral lobes trapezoidal and extended caudally. Medioventral process triangular in ventral view, apical margin rounded. Anal segment (Figs 9, 11) tubular, dorsal margin almost straight, ventral margin waved in lateral view; 2.6 times longer than wide in dorsal view; anal style strap-shaped, not beyond anal segment. Genital styles (Figs 10, 12) symmetrical ventrally, inner margin with a small curved process near base; in lateral view, dorsal margin bending inwards medially, apical margin pediform. Aedeagus (Figs 13–16) with six processes. Right side of periandrium with a slender process, curved downwards basally and paralleled with periandrium distally; another short reversed spinose process directed dorsocaudally arising from basal 1/3 of right side of periandrium. Ventral margin with a large projection near apex, of which basal 1/3 longitudinally and distal 2/3 horizontally extended, directed ventrocephalically. Left side of periandrium with a very short spinose process, directed ventrocaudally. Flagellum with two processes, semi-sclerotized, generally curved in left side; apex with a very fine process, curved dorsocephalically; a process arising from base of flagellum, along the dorsal edge of the flagellum, directed ventrocephalically.

#### Distribution.

China (Yunnan) (Fig. 29).

#### Etymology.

The specific name is derived from the Latin adjective *long*- and *spinus*, referring to the one long process arising from the base of the flagellum, along the dorsal edge of the flagellum.

#### Remarks.

This species is similar to *Andixius nupta* Emeljanov & Hayashi, 2007 in appearance, but differs in: (1) right side of periandrium with a short reversed spinose process at apical 1/3 (*A. nupta* without process in the same position); (2) left side of periandrium with a small process, not furcate (left side of periandrium with a large bifurcate process in *A. nupta*); (3) flagellum with two processes (without process in *A. nupta*).

### Andixius nupta

Taxon classificationAnimaliaHemipteraCixiidae

Emeljanov & Hayashi, 2007

Andixius nupta Emeljanov & Hayashi, 2007: 128–130, figs 1, 8–13.

#### Distribution.

Japan (Ryukyu) (Fig. 29).

#### Remarks.

Based on the description and the figures by Emeljanov and Hayashi (2007), this species can be distinguished from the other species of the genus by the following characters: left side of periandrium with a bifurcate process medially; left-ventral margin with a reversed process and right-ventral margin with a long process apically.

### Andixius trifurcus

Taxon classificationAnimaliaHemipteraCixiidae

Zhi & Chen sp. n.

http://zoobank.org/C1B50C3D-4399-4ED0-BCDC-C4870F268D1D

Figs 3–4 , 17–28

#### Type material.

Holotype: ♂, **China**: Yunnan, Lushui County, Pianma Town (26°N, 98°36'E), 17–19 June 2011, Jian-Kun Long; paratypes: 4♂♂5♀♀, same data as holotype, Jian-Kun Long, Yu-Jian Li; same collecting site as holotype, 14 August 2006, Pei Zhang.

#### Description.

Body length: male 6.4–6.8mm (*N* = 5), female 7.9–8.2mm (*N* = 5); forewing length: male 5.4–5.9 mm (*N* = 5), female 7.1–7.3 mm (*N* = 5).

*Coloration.* General color yellowish brown (Figs 3–4). Eyes brown, ocelli faint yellow, semi-translucent. Antenna blackish brown. Vertex generally blackish brown with two short longitudinally yellow strips. Face generally brown. Postclypeus yellowish brown, rostrum yellowish brown except for apex dark brown. Pronotum with discal area light yellowish brown and lateral areas yellowish brown. Mesonotum brown. Forewing similar to *Andixius longispinus* sp. n., but without a tan spot near claval fork and distal half of forewing with larger brown patches. Hind tibiae and ventral abdomen yellowish brown.

**Figures 17–28.**
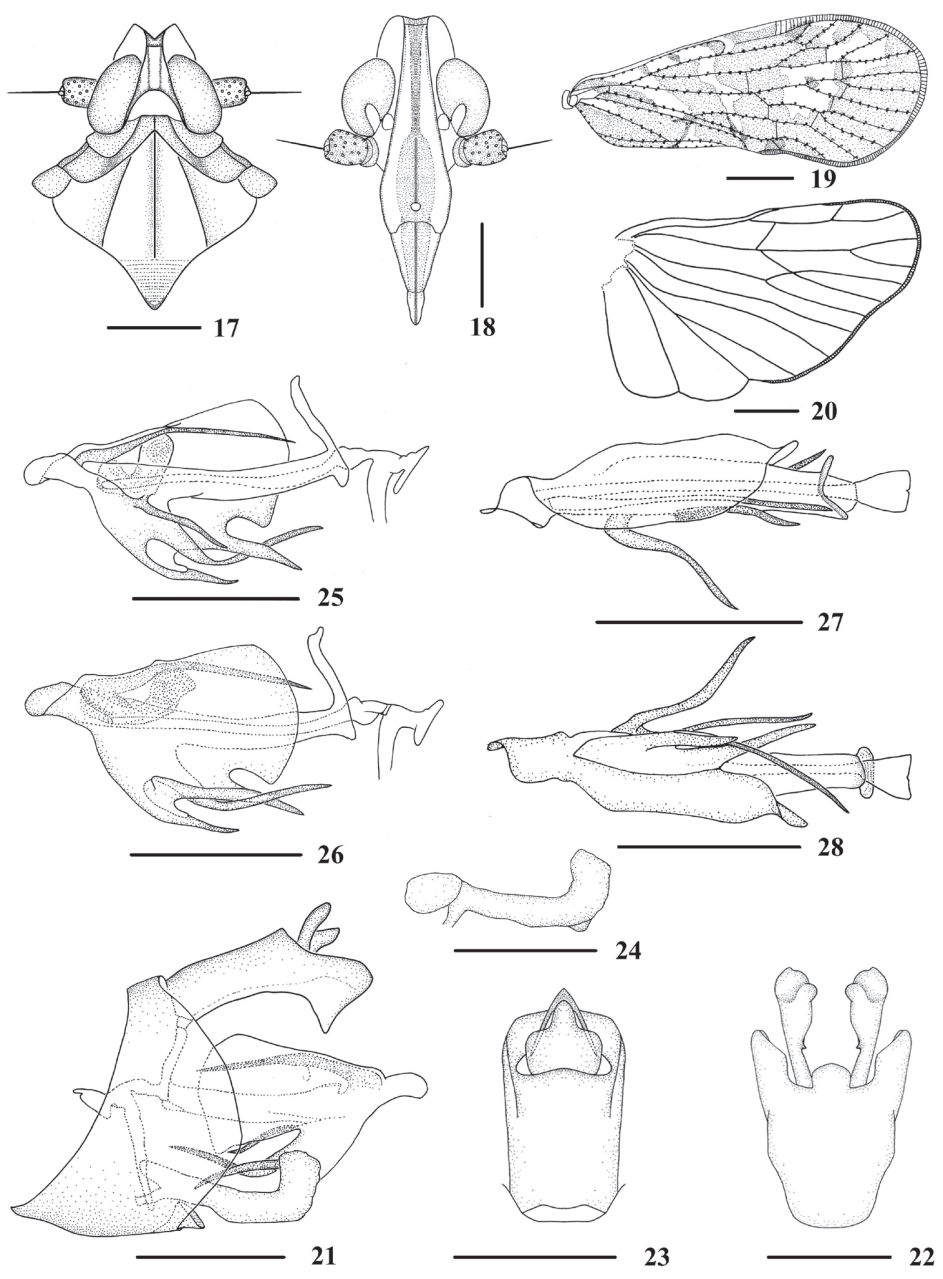
*Andixius trifurcus* sp. n., male **17** Head and thorax, dorsal view **18** Face, ventral view **19** Forewing **20** Hindwing **21** Genitalia, lateral view **22** Pygofer and genital styles, ventral view **23** Anal segment, dorsal view **24** Genital styles, lateral view **25** Aedeagus, right side **26** Aedeagus, left side **27** Aedeagus, dorsal view **28** Aedeagus, ventral view. Scale bars: 0.5 mm (**17–18, 21–28**); 1.0 mm (**19–20**).

*Head and thorax*. Vertex (Figs 3, 17) almost equal to width; anterior and posterior margin recessed in acute angle, median carina absent. Frons (Fig. 18), 2.6 times as long as wide. Pronotum (Figs 3, 17) 1.5 times longer than vertex; posterior margin recessed in a right angle. Mesonotum 1.4 times longer than pronotum and vertex combined. Forewing (Figs 4, 19) 2.3 times longer than wide, with twelve apical cells and seven subapical cells. Hind tibia with six lateral spines, chaetotaxy of hind tarsi: 6/6, 2^nd^ hind tarsus with two platellae.

*Male genitalia*. Pygofer (Figs 21–22) symmetrical, dorsal margin shallowly concave and U-shaped ventrally, widened towards apex; in lateral view, lateral lobes trapezoidal and extended caudally. Medioventral process round in ventral view. Anal segment (Figs 21, 23) with dorsal margin nearly straight, ventral margin with an antler-like process extending to apex ventrally in lateral view; 1.6 times longer than wide in dorsal view; anal style strap-shaped, slightly beyond anal segment. Genital styles (Figs 21, 24) symmetrical ventrally, inner margin with a small odontoid process medially and an obtuse process near apex, gradually widened towards apex; dorsal and ventral margins subparallel, apical part strongly bent upward and apical margin truncated in lateral view. Aedeagus (Figs 25–28) with five large processes. Dorsal margin of aedeagus near apex with a long process, slightly directed ventrocephalically. Periandrium with an expanded semi-enclosed structure around the left side and ventral margin of periandrium, ventral margin of the expanded structure with three long processes: apical one wide, slightly curved and directed cephalically; middle one longest, narrowed from base to end, curved upwards and directed dorsocephalically; basal one wide, slightly curved and directed ventrocephalically. A slender process arising from apical 1/3 of left side of periandrium, directed ventrocephalically. Flagellum short and small, slightly sclerotized, without process.

#### Distribution.

**Figure 29.**
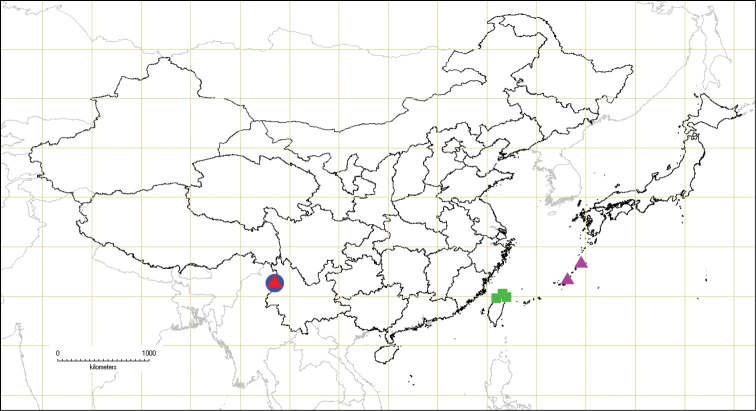
Geographic distributions of *Andixius* species: *A. longispinus* sp. n. (▲); *A. nupta* (▲); *A. trifurcus* sp. n. (●); *A. venustus* (■).

China (Yunnan) (Fig. 29).

#### Etymology.

The specific name is derived from the Latin prefix *tri*- plus the Latin word *furcus*, referring to the trifurcated ventral margin of the periandrium.

#### Remarks.

This species is similar to *Andixius longispinus* sp. n. in appearance, but differs in: (1) dorsal margin of aedeagus with a long process near apex (*A. longispinus* without process in the same position); (2) periandrium with an expanded semi-enclosed structure around left side and ventral margin of periandrium (not as above in *A. longispinus*); (3) flagellum without process (two processes in *A. longispinus*).

### Andixius venustus

Taxon classificationAnimaliaHemipteraCixiidae

(Tsaur & Hsu, 1991) in Tsaur, Hsu & Stalle, 1991

Brixia venusta Tsaur & Hsu, 1991, in Tsaur et al. 1991: 66, fig. 33 (A–I).
Andixius venustus (Tsaur & Hsu, 1991): Emeljanov and Hayashi 2007: 129.

#### Distribution.

China (Taiwan) (Fig. 29).

#### Remarks.

Based on the description and the figures by Tsaur & Hsu, 1991, *Andixius venustus* closely resembles *A. nupta* Emeljanov & Hayashi, 2007, but can be distinguished from the latter by the following characters: (1) right side of flagellum with a large bifurcate process basally (flagellum without process in *A. nupta*); (2) left side of periandrium with a medium process apically and without process medially (*A. nupta* without process apically and with a bifurcate process medially).

## Supplementary Material

XML Treatment for Andixius

XML Treatment for Andixius longispinus

XML Treatment for Andixius nupta

XML Treatment for Andixius trifurcus

XML Treatment for Andixius venustus
